# Validation of γ‐radiation and their effect on phenolic compounds, antioxidant activity, and microbial load of fennel (*Foeniculum vulgare*) seeds and cinnamon (*Cinnamomum verum*) sticks

**DOI:** 10.1002/fsn3.3233

**Published:** 2023-01-27

**Authors:** Salma M. Ahmed, Amro B. Hassan

**Affiliations:** ^1^ Sudanese Atomic Energy Commission (SAEC) Khartoum Sudan; ^2^ Environment and Natural Resources and Desertification Research Institute (ENDRI), National Center for Research Khartoum Sudan; ^3^ Department of Food Science and Nutrition, College of Food and Agricultural Sciences King Saud University Riyadh Saudi Arabia

**Keywords:** antioxidants, irradiation, microbial load, phenolic, PLS, spices

## Abstract

The aim of this study was to validate the optimum doses of γ‐radiation and its effect on the antioxidant capacity and microbial load of fennel seeds and cinnamon sticks. Gamma irradiation was applied in different doses 0.0, 2.5, 5.0, 7.5, 10, and 15 kGy. The findings stated that increasing gamma dose significantly (*p* < .05) increased the total phenolic content of the fennel seeds; however, it was decreased in cinnamon at doses higher than 5 kGy. The total flavonoid content was found higher after treatments at 5 kGy or more minor. After the gamma irradiation treatments, the antioxidant activities were enhanced. The microbial load of these spices was reduced after treatment. Doses more than 10.0 kGy are required to lower the bacterial load in samples, while only 5.0 kGy is sufficient to eliminate fungi growth. The partial least squares regression analysis stated the application of 7.5 kGy and reflects the most valid treatment doses for radiation treatments of fennel seeds and cinnamon sticks. Accordingly, it can be discovered that the γ‐radiation at a dose of 7.5 kGy could be considered a suitable dose for the preservation and decontamination of these spices and also for enhancing its antioxidant capacity. Three spices were subjected to gamma irradiation at different doses. The application of gamma radiation significantly reduces the level of the microbial load in the spices. Gamma irradiation improved the antioxidant capacity of the spices. Gamma irradiation can be applied as an effective preservative method in the food industries.

## Graphical abstract

Three spices were subjected to gamma irradiation at different doses. Application of gamma radiation significantly reduces the level of the microbial load in the spices. Gamma irradiation improved the antioxidant capacity of the spices. Gamma irradiation can be applied as an effective preservative method in the food industries.

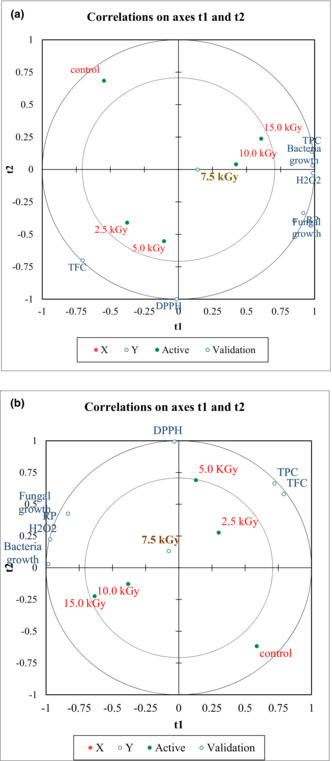

## 1 INTRODUCTION

Spices are dried seeds, used mainly for their unique sensory characteristics such as flavor, colors, and aroma to enhance the palatability of food worldwide. Also, they are rich in phenolic compounds, which serve as solid antioxidants (Pizzale et al., 2002; Su et al., 2007). Nevertheless, throughout the postharvest handling, they might be exposed to microbial infection. Such infection may occur due to unsafe environmental situation and miss‐handling practices under which they are processed and stored (Eliasson et al., 2015). In addition, since most spices are dried conventionally in the open air, applying these drying methods may result in high contaminated spices with air‐ and soil‐borne bacteria, fungi, and insects (Eliasson et al., 2015). Therefore, reducing the number of spoilage microorganisms and eliminating pathogens in spices is required.

It has been reported that bacterial plate counts of one to 100 million per gram in spice are usual (Bendini et al., 1998). However, following good manufacturing practices during the harvesting and processing of spices should improve their hygienic quality to acceptable microbiological and purity levels, as suggested by WHO (1999). Rtmitchell (2003) found that most of the spices, either whole or grounded, are contaminated with heat‐resistant bacterial spores and molds, and the contamination level ranged from 10^3^ to 10^8^ CFU/g. Moreover, it was reported that spices might contain pathogenic microorganisms and toxigenic molds considered a potential hazard to human health (Phianphak et al., 2007). However, the microbial characteristics of spices vary based on their origin and postharvest processing and storage condition (Mandeel, 2005).

Commonly, the conventional decontamination methods are not appropriate for spices disinfection. The application of traditional heating in spices may destroy their quality. On the other hand, extreme application of chemical fumigants is being progressively omitted in many countries since they might harm human health and the environment (Fowles et al., 2001). Therefore, gamma radiation has been explored to decontaminate the biological contaminants in food.

Gamma irradiation of food is generally used, and its application has been reviewed. Conversely, several studies have stated the effects of gamma radiation, particularly at high doses, on the quality of products through oxidation such as the reduction in levels of phytochemical compounds in black pepper and rosemary (Calucci et al., 2003), decreasing the antioxidant capacity of rosemary, cumin, thyme, and black pepper (Gumus et al., 2011; Kim et al., 2009; Pérez et al., 2007; Suhaj et al., 2006). Thus, the objectives of this study are to investigate the minimum effective and the most valid and optimum gamma dose that impacts antioxidant capacity and microbial loads of fennel seeds and cinnamon sticks.

## 2 MATERIALS AND METHODS

### 2.1 Sample collection and preparation

Two different dried spices (fennel seeds and cinnamon sticks) were collected from the local market. Samples were carefully cleaned and freed from foreign materials, ground using sterile laboratory miller, and then packed in polyethylene bags.

### 2.2 Gamma radiation treatment

Gamma radiation treatment was done at the Kaila irradiation processing unit, Sudanese Atomic Energy Corporation (SAEC), according to Hassan et al. (2019). Spice samples were exposed to incremental doses (2.5, 5.0, 7.5, 10, and 15 kGy) of gamma radiation. Un‐irradiated spices were defined as controls (0 kGy), and three replicates of each treatment were performed.

### 2.3 Extraction of antioxidants

The methanolic extract of the spice samples was prepared at a ratio of 1:25 (w/v) at 25 °C overnight. The extract was collected, and the process was repeated for the residue. The collected extracts were dried using a vacuum and a rotary evaporator and kept for further analysis.

### 2.4 Determination of total phenolic content

The total phenolic content (TPC) of fennel seeds and cinnamon sticks was measured according to Waterhouse (2002). To 1.58 ml of H_2_O and 100 μl of the Folin–Ciocalteu reagent, 20 μl of the extract solution and 300 μl of Na_2_CO_3_ were vortexed (1 min) and kept at 20°C for 2 h. The absorbance was detected at 765 nm in contradiction to the blank solution. A calibration curve was conducted with different concentrations of gallic acid (*R* ^2^ = 0.9672), and the results are described as milligram Gallic Acid Equivalents GAE/g sample (DW).

### 2.5 Determination of total flavonoid content

The total flavonoid content (TFC) of the spices was estimated by following the method of Kim et al. (2003). An aliquot (1 ml) solution was prepared from the dried methanolic extract (1:10 w/v), 5% NaNO_2_ solution (300 μl) and 10% AlCl_3_ (300 μl) mixed and incubated for 5 min. Then, about 2 ml of the NaOH (2 N) was added and the volume of the mixture was filled with H_2_O to 10 ml, and the absorbance was detected at 510 nm. A calibration curve was completed from different concentrations of catechin (*R* ^*2*^ = 0.974), and the total flavonoid content is expressed as milligram Catechin Equivalents (C.E.)/g sample (DW).

### 2.6 Antioxidant activity

#### 2.6.1 Diphenyl‐2‐picrylhydrazyl scavenging assay

The scavenging activity of diphenyl‐2‐picrylhydrazyl (DPPH) radicals of the radiated and un‐radiated spice extracts was performed (Chang et al., 2001). A mixture of the extract (100 μl) or deionized H_2_O (as a control), 50 mM Tris–HCl buffer pH 7.4 (900 μl), and 1000 μl DPPH were incubated at room temperature for 30 min. The absorbance was then determined at 517 nm. DPPH scavenging was calculated according to the following formula:

$$\begin{array}{c} \text{DPPH scavenging}\;\left(\%\right)\\ =\left[\left(\text{Absorbance control}–\text{Absorbance sample}\right)/\text{Absorbance control}\right]\times 100 \end{array}$$

#### 2.6.2 Reducing power

The reducing power (RP) of the samples was determined by following the method of Gulcin et al. (2002). Briefly, 2.5 ml of phosphate buffer (0.2 M, pH 6.6) and 2.5 ml K_3_[Fe(CN)_6_] (1%) were added to the extract. After incubation (50°C for 20 min), about 2.5 ml of TCA (10%) was added to the mixture and centrifuged at 1038 × *g* for 10 min. After that, 2.5 ml of the supernatants was mixed with 2.5 ml of H_2_O and 0.1% FeCl_3_ (0.5 ml). The absorbance was detected at 700 nm. Ascorbic acid is employed as a standard antioxidant. Total reducing power was expressed as ascorbic acid equivalents (AAE/g sample).

#### 2.6.3 Hydrogen peroxide scavenging assay

A hydrogen peroxide (H_2_O_2_) scavenging assay was determined according to Jayaprakasha et al. (2004). An H_2_O_2_ (40 mM) solution was prepared in phosphate buffer (pH 7.4). Approximately 1 mg/mL of the extract was prepared and mixed with 3 ml of phosphate buffer, and then 1 ml of H_2_O_2_ (40 Mm) was added. After incubation for 10 min, the absorbance was collected at 230 nm. The H_2_O_2_ scavenging ability was calculated as follows:

$$H_{2}O_{2}\;\text{scavenging ability of samples}\;\left(\%\right)=\left\{\left(\mathrm{Ab}-\mathrm{As}\right)/\mathrm{Ab}\right\}\times 100$$

where Ab is the absorbance of the control and As is the absorbance of the sample.

### 2.7 Microbiological examinations

The microbiological examinations of radiated and nonradiated spice types included the determination of the total viable count of bacteria (CFU/g) and the fungal incidence and colony formation (CFU/g). One gram of each sample was homogenized in 10‐ml peptone water (0.1%) using vortex apparatus for 1–2 min to give a final dilution of 1:10. Liquid samples were serially diluted and plated using the appropriate medium.

#### 2.7.1 Total count of bacteria determination

The total count of bacteria with a serial dilution of 10^−3^ was plated on plate count agar (PCA) medium according to APHA (1993). After incubation at 37 ± 2°C for 48 h, the colonies were counted, and the total bacterial counts were expressed as colony‐forming units (CFU) per gram of samples.

#### 2.7.2 Fungal culture and incidence

The fungal growth in treated and untreated spices was evaluated as colony‐forming unit per gram (CFU/g) according to the AOAC (2005) methods. From each sample dilution, 1 ml was platted on Potato Dextrose Agar (PDA) and then incubated at 25 ± 2°C for 5 days.

### 2.8 Statistical analysis and validation

All data were the mean of triplicate. Data were analyzed using a one‐way analysis of variance (ANOVA). Significant differences were calculated (*p* < .05) using the least significant difference (LSD). In addition, a linear partial least squares regression test (PLS) was performed to validate and optimize gamma dose using the XLSTAT software (Tenenhaus et al., 2005).

## 3 RESULTS AND DISCUSSION

### 3.1 Effect of gamma irradiation treatment on total phenolic content and total flavonoid content of fennel seeds and cinnamon sticks

Table 1 shows the effect of gamma radiation on the total phenolic content (TPC) of fennel seeds and cinnamon sticks. Before radiation process, the TPC of fennel and cinnamon was found to be 52.5 and 26.6 mg GAE/g DM, respectively. Gamma radiation caused a significant (*p* < .05) effect on the TPC of all spices. With an increase in gamma doses, a significant (*p* < .05) increment in the TPC was observed for the fennel seeds, and the highest TPC value, 65.5 mg GAE/g DM, was obtained when exposed to a 15.0 kGy. However, for cinnamon, the highest TPC value was received at 5.0 kGy. Increasing the gamma dose to 7.5, 10, and 15 kGy caused a significant (*p* < .05) reduction in the TPC of the cinnamon to 27.9, 25.9, and 24.2 mg GAE/g DM, respectively.

**TABLE 1:** Effect of gamma radiation on total phenolic content (TPC) and total flavonoid content (TFC) of fennel seeds and cinnamon sticks

Spices	Gamma radiation (kGy)	TPC (mg GAE/g)	TFC (mg CE/g)
Fennel seeds	Control	52.5 ± 1.14^d^	65.6 ± 0.14^c^
	2.5	52.1 ± 1.29^d^	94.5 ± 2.50^a^
	5.0	56.0 ± 1.29^c^	84.9 ± 3.82^b^
	7.5	62.1 ± 1.28^b^	66.6 ± 1.46^c^
	10.0	65.4 ± 1.25^a^	57.0 ± 3.31^d^
	15.0	65.5 ± 0.55^a^	48.3 ± 3.31^e^
Cinnamon sticks	Control	26.6 ± 0.25^c^	25.5 ± 0.14^c^
	2.5	27.7 ± 0.14^b^	29.4 ± 0.36^a^
	5.0	28.4 ± 0.20^a^	28.9 ± 0.07^b^
	7.5	27.9 ± 0.29^b^	22.0 ± 0.38^d^
	10.0	25.9 ± 0.25^d^	20.0 ± 0.40^e^
	15.0	24.2 ± 0.29^e^	19.7 ± 0.31^e^

*Note*: Data represent the mean ± SD (*n* = 3). Values followed by the same letter are not significantly different (*p* < .05) as assessed by LSD.

In assumption, the principal finding of the present study is that gamma radiation concomitantly improved the TPC of these spices and increased gradually with the rises in the gamma dose with an exceptional in cinnamon it gradually decreased after 5 kGy. Obtained results agree with Ashouri Sheikhi et al. (2016), who found that increasing gamma doses significantly increased the TPC of Galbanum. Moreover, Jamshidi et al. (2014) observed that the TPC of *Echinacea purpurea* was increased after radiation treatment. Another increase in the total phenolic contents in pomegranate peels after gamma radiation up to 10 kGy was stated by Mali et al. (2011).

Conversely, some authors reported a reduction in some spices' phenolic compounds, particularly after exposure to high gamma rays. Koseki et al. (2002) showed that the TPC of dehydrated rosemary sharply decreased after radiation with doses >10 kGy. Comparable loss in the TPC of *Thymus vulgaris* and *Thymbra spicata* extracts was also observed by Gumus et al. (2011). However, Brandstetter et al. (2009) reported that the total phenolic content of sage, thyme, and oregano did not change after gamma radiation treatment.

In this study, the increases in TPC on fennel and cinnamon after radiation treatment could be a result of the collapse of high‐molecular‐weight phenolic compounds to smaller ones, which may contribute to the increase in the TPC after gamma irradiation (Sádecká, 2007). However, the reduction in TPC of cinnamon after high doses of treatment might be due to the degradation of phenolic compounds by radiation. Anna (2013) and Park et al. (2009) stated that the degradation of phenolic compounds leads to leach out of the phenolic compound during the preparation of the extract.

The effect of gamma irradiation on the total flavonoid content (TFC) of the fennel seeds and cinnamon‐dried powder is highlighted in Table 1. In general, the TFC of the spices was significantly (*p* < .05) affected by gamma radiation. The high doses of gamma (>5 kGy) caused a significant (*p* < .05) reduction in the TFC of both spices. The highest value of the TFC in fennel (94.5 mg CE/g) and cinnamon (29.4 mg CE/g) was noted when they were treated with gamma radiation at a dose of 2.5 kGy. However, rising the gamma dose from 5 kGy to 15 kGy slightly decreased the TFC of both fennel seeds and cinnamon sticks.

Khatun et al. (2017) found that radiation treatment and increasing radiation dose reduced the TFC of garlic and turmeric whereas showed an increase in ginger. Carocho et al. (2012) and Shahzad et al. (2017) showed that gamma radiation decreased flavonoid content in chestnut, black cumin, and *C. tuberculata*. In converse, Pereira et al. (2016) reported that irradiated thyme at 10 kGy increased its TFC.

Differences in the TFC in these spices impacted by gamma irradiation might also be due to the variances in the spice types, and the decrease in TFC might be attributed to the degradation of flavonoid compounds by gamma irradiation at high doses and loss of them during extract preparation (Anna, 2013).

### 3.2 Effect of gamma irradiation treatment on the antioxidant activity of spices of fennel seeds and cinnamon sticks

In this study, the antioxidant activity of the fennel seeds and cinnamon sticks was determined by using different methods in terms of DPPH scavenging activity, reducing power, and hydrogen peroxide scavenging activity (Table 2).

**TABLE 2:** Effect of gamma radiation on DPPH scavenging activity, reducing power (RP), and hydrogen peroxide scavenging (H_2_O_2_) of fennel seeds and cinnamon sticks

Spices	Gamma radiation (kGy)	DPPH (%)	RP (mg AAE/g)	H_2_O_2_ (%)
Fennel seeds	Control	79.0 ± 1.27^c^	3.7 ± 0.08^d^	67.7 ± 0.15^f^
	2.5	86.4 ± 0.31^a^	4.2 ± 0.05^c^	71.1 ± 0.42^e^
	5.0	87.4 ± 1.42^a^	4.2 ± 0.08^c^	74.1 ± 0.22^d^
	7.5	83.2 ± 0.16^b^	4.4 ± 0.06^b^	75.7 ± 0.05^c^
	10.0	83.8 ± 0.00^b^	4.5 ± 0.06^b^	79.6 ± 0.14^b^
	15.0	82.3 ± 0.85^b^	4.6 ± 0.06^a^	84.8 ± 0.18^a^
Cinnamon sticks	Control	75.8 ± 0.17^c^	25.5 ± 0.14^e^	93.5 ± 0.14^f^
	2.5	77.9 ± 0.93^ab^	26.9 ± 0.08^d^	94.4 ± 0.10^e^
	5.0	78.7 ± 1.42^a^	27.2 ± 0.15^c^	94.6 ± 0.07^d^
	7.5	77.1 ± 0.57^bc^	27.4 ± 0.09^c^	94.9 ± 0.06^c^
	10.0	76.8 ± 0.28^bc^	27.8 ± 0.14^b^	95.2 ± 0.05^b^
	15.0	76.9 ± 0.00^bc^	28.2 ± 0.08^a^	95.5 ± 0.13^a^

*Note*: Data represent the mean ± SD (*n* = 3). Values followed by the same letter are not significantly different (*p* < .05) as assessed by LSD.

The DPPH scavenging activity of the fennel seeds and cinnamon sticks was found to be 79% and 75.8%, respectively. In general, gamma rays significantly (*p* < .05) increased the DPPH scavenging activity of spices. The highest values, 87.4%, 86.4%, 78.74%, and 77.88%, of DPPH scavenging activity were achieved when fennel and cinnamon were treated with 5 kGy and 2.5 kGy, respectively. However, increasing the dose gradually to 15 kGy significantly (*p* < .05) decreased their DPPH scavenging activity to 82.3% and 76.94%.

The estimation of the capability of the antioxidant to donate an electron is required. Gulcin (2020) mentioned that reducing power assay is frequently used to assess the reduction of Ferric ions. Nonirradiated samples significantly (*p* < .05) had the lowest decreasing power (RP) values compared to irradiated samples. As shown in Table 2, increasing the gamma dose caused a significant (*p* < .05) increase in the RP value of fennel seeds and cinnamon sticks. The highest RP activity of 6.62 and 4.61 mg AAE/g was noticed for the 15 kGy dose in fennel and cinnamon, respectively. Likewise, the increase of RP activity after the radiation process was observed by Rezanejad et al. (2019). They reported that the RP activity of rosemary extract increased with increasing absorbed doses and reached the maximum at the dose of 30 kGy. Also, Rajurkar et al. (2012) stated that radiation treatment increased the reducing power of *Justicia adhatoda*.

Hydrogen peroxide (H_2_O_2_) is considered one of the specific radicals involved in oxidative stress. Hence, natural antioxidants scavenging these radicals are essential to protect the biological systems (Gulcin, 2020). As shown in Table 2, the H_2_O_2_ scavenging capacity of the control fennel seeds and cinnamon sticks was 67.7% and 93.5%, respectively. The application of gamma radiation caused a significant (*p* < .05) increment in the H_2_O_2_ scavenging activity of fennel seeds and cinnamon sticks. The proliferation of H_2_O_2_ scavenging capacity shows a gradual pattern as the dose increases. The highest activity values were obtained when spices were treated with a gamma dose of 15 kGy. Our findings align with the previous study reported by El‐Shora et al. (2015), which stated that the H_2_O_2_ scavenging was significantly enhanced after gamma radiation treatment of fenugreek. It was progressively increased with the increasing gamma doses.

Despite the variation between the antioxidant assays, in this study, the DPPH, R.P, and H_2_O_2_ assays showed high antioxidant activity for fennel and coriander, particularly after radiation treatments.

### 3.3 Effect of gamma irradiation on the total count of bacteria and fungi in different types of spices

Total bacterial count is the most common quality parameter for assessing the hygienic status of food and food additives. Table 3 shows the total bacteria count of fennel and cinnamon before and after radiation treatments. As shown in the table, the total count of bacteria (*p* < .05) was significantly influenced by radiation dose in all spices. Before radiation treatment, the total number of bacteria was found to be 6.6 and 6.4 log CFU/g in fennel and cinnamon, respectively. The total count of bacteria was significantly (*p* < .05) decreased as the radiation dose increased. The maximum growth inhibition of the bacteria was observed at the high radiation dose of 15 kGy. After that level, it reached 100% fennel and cinnamon, respectively (Table 3).

**TABLE 3:** Effect of gamma radiation on the bacterial growth (TVC) on fennel seeds and cinnamon sticks

Spices	Gamma radiation (kGy)	TVC (Log CFU/g)	Growth inhibition (%)
Fennel seeds	Control	6.6 ± 0.00^a^	–
	2.5	6.4 ± 0.06^b^	3.9 ± 0.10
	5.0	6.3 ± 0.07^b^	4.2 ± 0.11
	7.5	6.3 ± 0.01^b^	5.0 ± 0.17
	10.0	5.8 ± 0.05^c^	12.8 ± 0.31
	15.0	5.5 ± 0.04^d^	16.1 ± 0.68
Cinnamon sticks	Control	6.4 ± 0.01^a^	–
	2.5	4.2 ± 0.21^b^	34.4 ± 3.36
	5.0	4.2 ± 0.21^a^	34.4 ± 3.36
	7.5	4.2 ± 0.21^b^	34.4 ± 3.36
	10.0	2.0 ± 0.00^c^	68.9 ± 0.40
	15.0	0.0 ± 0.00^d^	100 ± 0.00

*Note*: Data represent the mean ± SD (*n* = 3). Values followed by the same letter are not significantly different (*p* < .05) as assessed by LSD.

The effect of gamma radiation on fungal growth is highlighted in Table 4. The table shows that untreated fennel samples are highly contaminated with fungi compared to cinnamon. It was found to be 5.4 and 4.0 log CFU/g in fennel and cinnamon, respectively. Gamma radiation of spices caused a significant (*p* < .05) reduction in fungal growth. A radiation dose of up to 5 kGy is required to eliminate fungi from fennel seeds and cinnamon sticks.

**TABLE 4:** Effect of gamma radiation on the fungal growth of fennel seeds and cinnamon sticks

Spices	Gamma radiation (kGy)	Fungi (Log CFU/g)	Growth inhibition (%)
Fennel seeds	Control	5.4 ± 0.04^a^	–
	2.5	4.0 ± 0.06^b^	26.4 ± 0.10
	5.0	0.0 ± 0.00^c^	100 ± 0.00
	7.5	0.0 ± 0.00^c^	100 ± 0.00
	10.0	0.0 ± 0.00^c^	100 ± 0.00
	15.0	0.0 ± 0.00^c^	100 ± 0.00
Cinnamon sticks	Control	4.0 ± 0.00^a^	–
	2.5	3.7 ± 0.00b	32.5 ± 0.00
	5.0	2.7 ± 0.02^c^	100 ± 0.0
	7.5	0.0 ± 0.21^d^	100 ± 0.0
	10.0	0.0 ± 0.00^d^	100 ± 0.0
	15.0	0.0 ± 0.00^d^	100 ± 0.0

*Note*: Data represent the mean ± SD (*n* = 3). Values followed by the same letter are not significantly different (*p* < .05) as assessed by LSD.

Spices used in this study obtained from a local market were found to be highly contaminated with bacteria and fungi. For all spices, bacterial contamination was found at more the 10^6^ CFU/g, which resulted from different pre‐ and postharvest factors. Radiation of fennel seeds and cinnamon sticks using Co ^60^ gamma source was found as an effective method to reduce the microbial load sharply. It was noted that a dose of 15 kGy or more was required to lower the total bacterial infection, sometimes less than detectable levels. In contrast, a dose of only 5 kGy was needed to eliminate the fungal contamination. According to Sádecká (2007), the sterilizing impact on germs in irradiated samples might be due to the damage of their DNA which often leads to cell death. Likewise, Shahzad et al. (2017) reported that gamma radiation treatment at doses of 1, 3, 5, and 7 kGy significantly reduced the bacterial and fungal load in garlic, ginger, *Caralluma tuberculata*, and black cumin. Moreover, Kirkin et al. (2014) reported that a gamma dose of more than 7 kGy eliminates thyme, rosemary, black pepper, and cumin.

The wide variations found in the microbiological quality of the fennel seeds and cinnamon sticks used in this study may be due to the general conditions during their cultivation, harvesting, and the postharvest handling. For example, the highest irradiation dose used (15.0 kGy) reduced the total bacterial count of cinnamon to less than 100 CFU/g. At the same time, it was still higher than the level recommended by WHO for other spices. However, Oh et al. (2003) stated that the effect of gamma irradiation on the microbial population of spices is dependent mainly on the type and the initial number of microorganisms present.

### 3.4 Partial least squares regression analysis

**FIGURE 1:**
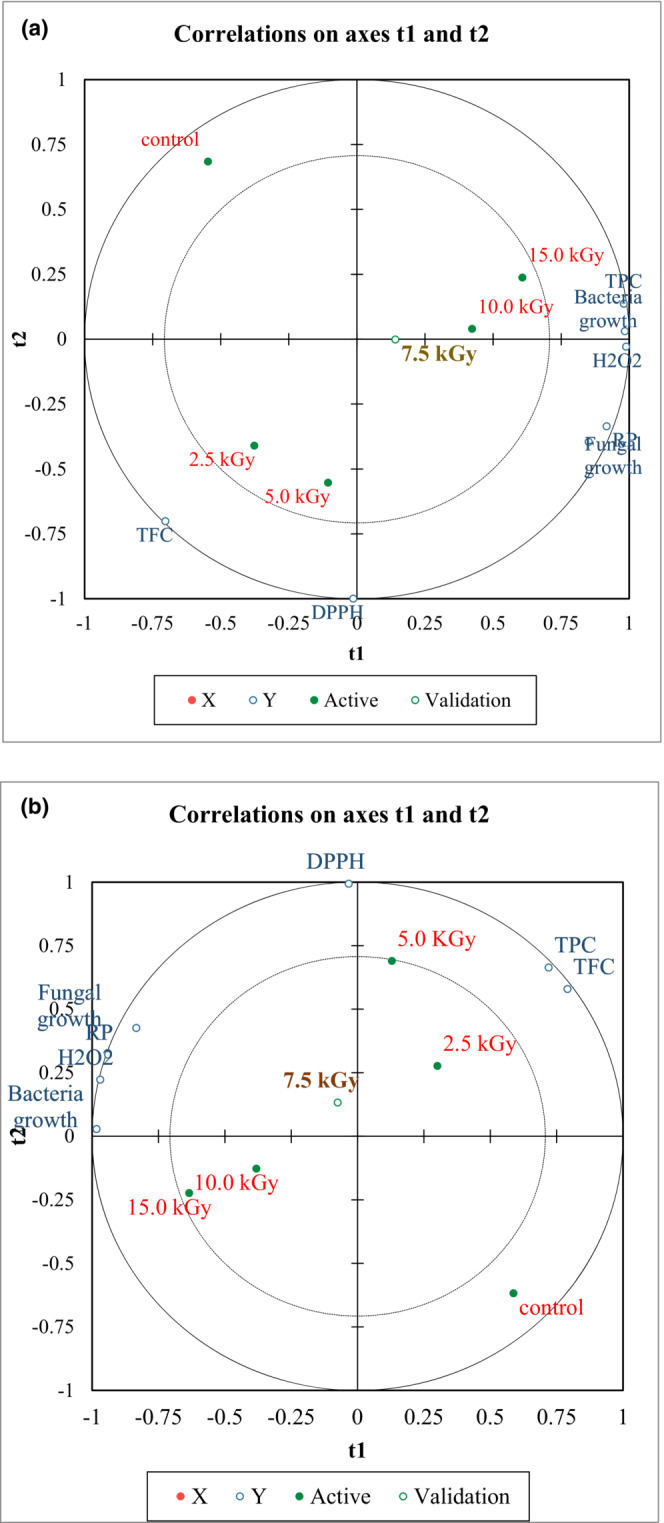
Partial least squares regression analysis (PLS) of the microbial load, antioxidant activity (DPPH, R.P, and H_2_O_2_), total phenolic content (TPC), and total flavonoid content (TFC) of gamma radiation treated and control fennel seeds (a) and cinnamon sticks (b).

The partial least squares regression analysis (PLS) described that the interactive effects of gamma irradiation treatments on the antioxidant capacity and microbial load of fennel seeds and cinnamon sticks were observed (Figure 1a,b). As described in Figure 1a, the radiation doses were grouped into three groups according to their impact on fennel seeds' microbial load and antioxidant capacity. The PLS revealed that the radiation treatments of fennel, particularly those treated with the doses 7.5, 10, and 15 kGy, showed a positive validation score for most of the studied parameters. However, the 7.5 kGy exhibits the most optimum and valid dose for most of the studied parameters.

According to the model in Figure 1b, the radiation doses were grouped into low and high doses regarding their effect on the microbial load and antioxidants of cinnamon sticks. The PLS revealed that the radiation treatments of cinnamon, particularly those treated with the doses 7.5 kGy, showed a positive validation score for most of the studied parameters. Thus, the PLS specified that the application of 7.5 kGy reflects the most proper treatment for fennel seeds and cinnamon sticks, which might consider for food industry applications.

## 4 CONCLUSION

This study explored to validate the dose of γ‐radiation to optimize gamma doses, enhancing antioxidant capacity and microbial load of fennel seed and cinnamon sticks. As a result, these treatments were found significantly enhance the content of the phenolic compounds and antioxidant activity of spices, particularly at low doses. Furthermore, the application of gamma radiation to contaminated spices significantly reduced their microbial load. Regarding the validation model, the application of 7.5 kGy shows great potential for its application in the food industry and could become an emerging effective postharvest preservative method to extend the shelf life of spices and enhance their antioxidant capacity.

## ACKNOWLEDGEMENTS

The authors are grateful to the Gamma Source Division of Sudanese Atomic Energy Corporation (SAEC), to provide their gamma irradiatorfacility.

## CONFLICT OF INTEREST

The authors declare that there is no conflict of interest.

## Data Availability

The data used to support the findings of this study are included within the article.
